# Effectiveness of Group Cognitive-Behavioral Therapy on Symptoms of Premenstrual Syndrome (PMS) ‎

**Published:** 2016-01

**Authors:** Maryam Maddineshat, Sodabe Keyvanloo, Hossein Lashkardoost, Mina Arki, Mahbubeh Tabatabaeichehr

**Affiliations:** 1 Department of Nursing, Nursing and Midwifery Faculty, Addiction and Behavioral ‎Sciences Research Center, North Khorasan University of Medical Sciences, Bojnurd, Iran; 2 Nursing Student of Research Committee, North Khorasan University of Medical ‎Sciences, Bojnurd, Iran; 3 Department of Public Health, Addiction and Behavioral Sciences Research Center, ‎North Khorasan University of Medical Sciences, Bojnurd, Iran; 4 Emam Reza Hospital, Bojnurd, Iran‎; 5 Department of Midwifery, Nursing and Midwifery Faculty, North Khorasan University of Medical Sciences, Bojnurd, Iran

**Keywords:** Cognitive-Behavioral Therapy, Group Therapy, Premenstrual Syndrome (PMS) ‎

## Abstract

**Objective: **Standards of care and treatment of premenstrual syndrome (PMS) vary. Non-drug ‎psychosocial intervention therapy is recommended for women with any kind of ‎discomfort or distress caused by PMS. The current study examined the effectiveness of ‎group cognitive-behavioral therapy on the symptoms of PMS at a girls’ dormitory of ‎North Khorasan University of Medical Sciences.

**Method:** In this quasi-experimental study, 32 female students with PMS who were majoring in ‎nursing and midwifery and residing in the dormitory were selected using the ‎convenience sampling method and were assigned to experimental and control groups. ‎The Standardized Premenstrual Symptoms Screening Tool was used as the research ‎tool. Eight sessions of cognitive-behavioral group therapy were held for the students

**Results:** There was a significant difference in psychological symptoms before and after ‎cognitive-behavioral therapy (p=0.012). Furthermore, cognitive-behavioral therapy was ‎effective on social interferences caused by PMS symptoms (p=0.012).‎

**Conclusion**: Group cognitive-behavioral therapy effectively alleviates PMS symptoms in female ‎college students.‎

Premenstrual syndrome, or PMS, is a universal phrase present in all cultures and has ‎been the subject of attention for many biomedical researchers (1). PMS is a disorder that ‎affects the lives of millions of women from menarche to menopause (2). This syndrome ‎consists of a wide range of physical, psychological, and behavioral symptoms that do ‎not result in any organic disease, occur regularly during the luteal phase of each ‎menstrual cycle, and resolve as menstruation ends. The severity and chronic nature of ‎PMS has led to the prevalence of this disorder in women and disrupts their work, ‎family relationships, and daily activities (3). Over 90% of women experience PMS in ‎their reproductive years. In addition, 10% experience premenstrual dysphoric disorder ‎‎ (PMDD), a severe form of PMS. (4, 5) In the United States, 28 million women suffer ‎from PMS, and 6 million are affected by PMDD (6). Ramezani Tehrani (2013) studied ‎‎18- to 45-year-old women in Iran between the years 2009 and 2011 and reported the ‎prevalence of PMS as 52.9%, of which 34.5% suffered from its severe form (7). Women ‎experience more than five hundred menstrual cycles during their reproductive years.‎‏ ‏These cycles have various symptoms, including depression; changes in mood, behavior, ‎and feeling of well-being (including mental image); food cravings; abdominal pain; ‎breast tenderness; headache; and fatigue. These symptoms intensify 4 to 7 days before ‎menstruation, and women are likely to suffer from these symptoms on particular day's ‎for 4 to 10 years. Like other major mood disorders, PMS and PMDD make a person ‎incapable of leading an organized life. Since PMS is associated with mental disorders, ‎there is a need for effective therapeutic interventions (6, 8). The Royal College of ‎Obstetricians and Gynecologists and the National Association for Premenstrual ‎Syndrome (NAPS) published clinical practical guidelines for the management of PMS ‎‎ (9, 10). In these guidelines, patients record their PMS symptoms for at least two or three ‎cycles in a questionnaire diary. Appropriate treatments are then selected based on the ‎recorded symptoms. Cognitive behavioral therapy is recommended in patients with ‎moderate to severe PMS and mood, emotional, and physical symptoms (11) ‎‏.‏

Cognitive-behavioral psychotherapy includes limited weekly sessions conducted by a ‎psychotherapist focusing on modifying negative and abnormal thoughts and training ‎individuals in effective adaptive mechanisms. The benefit of psychotherapy is that its ‎effects are achieved through time; even when psychotherapy sessions come to an end, ‎their effects remain. Conversely, pharmacotherapy has a very rapid effect, but it ends ‎with the discontinuance of the drug (12). Cognitive therapy focuses on cognitive ‎concepts, cognition related to the inner perception process, memory, and judgment with ‎which a person perceives herself and the world around her, and cognitive interventions ‎that help change or rectify individuals’ thinking patterns which have been shaped over ‎time. Such thinking patterns often interfere with individuals’ abilities and optimal ‎performance. The skills and techniques used in cognitive-behavioral therapy (CBT) are ‎models which investigate and determine the relationship between thoughts, feelings, ‎and behavior. In CBT, it is assumed that individuals are not disturbed by an event ‎itself; rather, it is the individual’s perception of that incident which is harmful. As long ‎as an individual believes something, she will nurture and develop that belief. Work and ‎practice can modify beliefs that create difficulties in life (13). In 2011, Nazari et al (14) ‎indicated that anxiety is the most common symptom of PMS. According to this study, ‎group CBT is able to free target individuals from distress and confusion through ‎planning and organizing, thus reducing anxiety. The individual avoids disturbing ‎thoughts that cause anxiety and comes to believe herself as a person who has certain ‎abilities and shortcomings. Moreover, avoiding hidden defects reduces the expectations ‎an individual has of herself and others. Results of this study showed that irritability is ‎reduced through CBT. Recent studies (15-17) have shown that a combination of ‎different treatments is much more effective than single treatment methods. Non-‎pharmacological treatment of chronic disorders is of particular importance, especially in ‎adolescents. One can greatly benefit from counseling techniques through which the ‎client is helped to identify his problem and, after becoming familiar with the unknown, ‎he proceeds to analyze his problem and choose a solution. Adolescence and youth are ‎the most common periods for the start of PMS symptoms. Given the negative effects ‎PMS has on psychological, physical, and mood dimensions of this critical period in the ‎life of girls, the first step for its prevention and for the promotion of physical and ‎mental health in these individuals is to increase their awareness by developing and ‎spreading extensive, organized programs. Based on the mentioned studies and ‎witnessing PMS symptoms among dormitory girls and their indiscriminate use of ‎medication, the current study was carried out with the purpose of reducing PMS ‎symptoms in female students in the dormitory of North Khorasan University of Medical ‎Sciences through group CBT.‎

## Materials and Method

In this quasi-experimental study was conducted at North Khorasan University of Medical ‎Sciences in the second semester of the 2011-2012 academic year. The study population was ‎female students living in the dormitory and majoring in nursing, midwifery, etc. Thirty-two ‎students with PMS were selected through non-probability convenience sampling upon acquiring ‎the Ethics Committee’s authorization (coded 90/p/380 on 10/2/2011) after the tenth meeting and ‎after obtaining informed consent from the participants. The sample was then divided into control ‎and experimental groups. Error type I was considered equal to 0.05 and error type II to 0.20 ‎‎ (P1:0.8, P2: 0.4). Thus, 16 individuals were selected for each group. The Premenstrual Symptoms ‎Screening Tool (PSST), originally developed in 2003 by researchers at McMaster University to ‎screen individuals with severe PMS or PMDD or those who may benefit from treatment, was ‎used in this study. This quick and reliable tool screening operationalizes classified DSM - IV ‎criteria (18-20). It‏ ‏has two parts. The first part consists of 14 items which measure emotional, ‎physical, and behavioral symptoms. The second part measures the impact of these symptoms on ‎the lives of individuals through 5 items, each of which has four options rated on a Likert scale - ‎not at all, mild, moderate, or severe symptoms - scored from 0 to 3. The following three ‎conditions should be met for the diagnoses of moderate to severe PMS: ‎

At least one of the first 4 items rated severe; ‎

In addition to the previous item, at least four items from questions 1 to 14 rated moderate ‎to severe; ‎

At least one of the five “functional” items (last 5 items) rated moderate or severe. Hariri et al. ‎evaluated the validity and reliability of this test in Iran with Tehran University of Medical ‎Sciences students who reside in dormitories. The reliability of the questionnaire obtained a ‎Cronbach’s alpha coefficient of 0.9. Content validity and content validity index values were 0.7 ‎and 0.8, respectively (21). First, the PSST was distributed among students. After individuals with ‎moderate to severe symptoms were identified, those with a score of 16 or higher were selected. ‎After obtaining informed consent, individuals were placed in experimental and control groups. ‎The experimental group was given explanations regarding their duties, rights, privileges, and ‎limitations. The experimental group received 8 to 12 ninety-minute group therapy sessions ‎which were held once a week from 8:00 to 9:30 pm in one of the classrooms at the School of ‎Nursing and Midwifery. The group therapy sessions took approximately 3 months to finish. ‎Members were approximately the same age and majoring in nursing or midwifery. Rights of the ‎members included:‎

Personal information should remain confidential and safe; ‎

Group members should have access to the group leader and assistant leader;‎

Members should be able to talk in private;‎

Members should not face any obligation for entering the group; ‎

Members have the right to actively participate in group activities.‎

In the recruitment stage, the leader and assistant therapist were careful to choose members who, ‎in addition to having the conditions of PMS, were interested in change and understood the ‎group’s objectives. The reason for their selection for group therapy was explained to individuals. ‎Members paid no tuition or any other costs for group therapy sessions. In each session, the ‎practice sheets and assignments were given to members free of charge. After finishing one ‎session and starting the next (with a week between the two), individuals had to note down their ‎actions and activities and bring it along with themselves to the next session. In group therapy ‎sessions, the group leader was not the only person to talk; rather, all members had to cooperate ‎and participate actively in the sessions. Each person talked about her experience with PMS, her ‎thoughts and feelings at the time, and the useful experience she had in encountering and ‎reducing the intensity of these symptoms intensity. Participants received training in at least 12 ‎cognitive-behavioral techniques throughout the course. During the last 30 minutes of each ‎session, the group leader gave a general summary of the issues discussed and gave the necessary ‎instructions for the assignments. Table 1 shows the treatment protocol. Data was analyzed using ‎statistical indicators along with Friedman statistical tests, and Wilcoxon signed-rank test were ‎used in SPSS version 19. ‎

## Results

This research was carried out on 27 female students who were eligible for the study. ‎This number included 14 individuals in the intervention group and 13 individuals in the ‎control group. Participants had a mean age of 21 years with a standard deviation of ‎‎0.79 and a median age of 21 years.‎‏ ‏Table 2 shows the significant improvement in ‎psychiatric symptoms experienced by the intervention group after intervention ‎compared with before intervention. No such change occurred in the control group. No ‎significant relationship was seen between the two groups in terms of psychological ‎symptoms before or after the intervention (Table 3). Table 4 shows that the symptoms ‎of social interferences were significant in both groups before and after the intervention ‎‎ (p<0.05), and Table5 shows no significant relationship between individuals’ ages and ‎the various conditions of PMS symptoms (p>0.05).‎

## Discussion

In this research, training was carried out with cognitive-behavioral group therapy based ‎on the principles of cognitive, emotional, and coping mechanisms. Treatment was based ‎on presenting information on such topics as relaxation, massage, nutrition, lifestyle ‎change, regular physical exercise, and stress management. The study’s findings show ‎that training with the group cognitive-behavioral therapy approach was effective on the ‎psychological symptoms of PMS in the experimental group. A significant difference ‎was seen between the trained experimental group and the control group. In addition, ‎the present study indicated that group cognitive-behavioral therapy is effective on the ‎social interferences caused by PMS; however, no significant effect was found on the ‎physical symptoms of PMS. These results were in line with similar studies carried out by ‎Christensen et al. in 1994 (22), Blake et al. in 1998 (23), Busse et al. in 2009 (24), Hofmann ‎et al. in 2012 [23], Taghizadeh et al. in 2010 (15), Hossein Nazari et al. in 2011 (14), and ‎Mirzai et al. in 2012 (25). These studies also revealed an improvement in some ‎psychological symptoms of PMS such as anxiety, depression, negative thoughts, and ‎physical changes. Furthermore, a 2011 study by Navabinejad et al. (16) examined the ‎effects of group cognitive-behavioral therapy on the physical symptoms of PMS in ‎married women. Its results suggested that CBT should be used to increase family ‎efficiency and reduce marital conflicts related to the menstrual period. The 2012 ‎findings of Mirzaie et al. (25) indicated that cognitive-behavioral stress management is ‎effective in reducing symptoms such as anxiety in women with PMS, but not on ‎physical symptoms. This study concluded that interventions in the form of drug therapy ‎and physical activity are able to reduce the physical symptoms of PMS. Since PMS ‎symptoms are caused by hormones and neurotransmitters, psychological treatments such ‎as stress management cannot prove effective in alleviating physical symptoms. In the ‎present study, the measurement of changes in physical symptoms was expressed ‎through a single question which students evaluated on a Likert scale. Thus, the ‎researchers believe that not being able to differentiate physical symptoms prevented ‎students from correctly evaluating and stating them. In investigating the frequency of ‎symptoms, a reduction before and after interventions was found. It cannot be definitely ‎asserted that cognitive-behavioral therapy has no effect on physical symptoms of PMS. ‎In addition, there is no single, accepted treatment across the globe for PMS. Previous ‎studies have not shown consistent results. Many clinical trials were not well-controlled ‎and have listed various methods for controlling the symptoms of PMS. It may be ‎helpful if the symptoms of stress are taken into consideration in psychological ‎counseling, such as cognitive-behavioral therapy or group therapy (23). In the current ‎study, the positive effects of cognitive-behavioral treatment on emotional and ‎psychological symptoms of PMS are justifiable. Based on the cognitive-behavioral ‎theory, disturbing thoughts and cognitive impairment lead to depression and stress. ‎PMS symptoms may be intensified and prolonged by the vicious cycle of negative ‎thoughts (low mood, low self-esteem, and lack of self-control) that women experience ‎in the premenstrual stage. One of the main strategies for making cognitive-behavioral ‎therapy more effective is to offer treatment in the form of group therapy (5, 26 and 27). In ‎the present study, the researchers tried to provide individuals with techniques for ‎identifying, challenging, and changing negative cognitions and inhibiting automatic ‎thoughts through 8 sessions of training. Carrying out the assignments was one of the ‎important aspects of this program. When a skill was presented with the assistant ‎therapist, individuals were expected to spend time at home working on other aspects of ‎their thinking and to use the proper technique in response to mood symptoms that ‎occur. The group leader set up each session in a way that members could express their ‎feelings and that enabled her to present participants with the necessary training. The ‎group leader had to establish a sense of security and confidence in individuals so that ‎they could self-disclose and express their feelings without anxiety. Cognitive behavioral ‎therapy was conducted with the assumption that students in dormitories are more ‎susceptible to environmental and emotional stressors such as economic difficulties, ‎special dorm circumstances, inconsistencies, and personal conflicts. These issues ‎intensify students’ mood symptoms. Since the participating students were majoring in ‎medical science fields, there was a higher possibility for self-medication (28). Although ‎the present study analyzed the effectiveness of group cognitive-behavioral therapy on ‎PMS symptoms, there was no follow-up on the group CBT’s lasting effects after the ‎completion of sessions, and the treatment method was not compared with other forms. ‎Hunter et al. (26) in 2002 studied the effectiveness of cognitive-behavioral therapy (ten ‎sessions) and Fluoxetine (20 mg) and combination therapy (Fluoxetine and cognitive ‎therapy) in women with premenstrual disorders. His results showed that a distinct ‎improvement with all three kinds of therapy had occurred after 6 months. In addition, ‎patients who received Fluoxetine experienced a quicker recovery. Follow-up on the ‎three groups showed no differences in terms of the betterment of premenstrual ‎disorders. However, it did reveal that cognitive-behavioral therapy had more lasting ‎effects than Fluoxetine therapy. To clarify, Fluoxetine and cognitive-behavioral therapy ‎both have equal treatment effects, but differ in terms of long-term effects. This can be ‎effective in deciding which type of treatment to choose. No added advantage was ‎observed in combination therapy. Ghaedi et al.’s (29) research in 2011 reported no ‎reduction in mean physical symptoms of the experimental and control groups after an 8-‎week follow-up, but mean psychological symptoms were significantly lower in the ‎experimental group than the control group. Thus, many studies (13, 22, 23and 25) suggest ‎that further experimental research must be carefully conducted with smaller groups ‎under controlled conditions in order to devise a tool that can assess the experiences of ‎women with PMS-related disorders. ‎

**Table 1:** Cognitive behavioral therapy approach in managing symptoms of premenstrual syndrome

**Session 1**	Introduction of therapist and group members. Students filled out questionnaires. Therapist gave a description of PMS; elimination of signs and symptoms; therapeutic agents; outcomes and impacts; the role of environmental stressors and psychological factors. **Assignment**: Fill out forms on PMS
**Session 2**	Description of group therapy, cognitive-behavioral interventions, duties and norms; description of group meeting to increase motivation and group cohesion; description of group objectives, determining behavioral changes, encountering initial anxiety of group members; opening up resistance and promoting self-disclosure. **Assignment**: Record moods one week before start of menstrual cycle, and note down physical changes caused by moods.
**Session 3**	Evaluation of automatic thought and cognitive distortions; emotional reasoning, generalized extreme, discussion and analysis of anxiety, depression, and symptoms that occur periodically. Reviewing the basis of specific experiences by retaining confidentiality and providing feedback. **Assignment**: Detect thoughts, moods and behaviors experienced in different life situations and identify which parts of those experiences need to be changed.
**Session 4**	Training members on emotional reactions and their relation to internal dialogue; exploring situations; having a broader and more objective perspective; dealing with resistance, tension, and anxiety. **Assignment**: Consider a recently experienced mood, identify moods during or immediately after it, and observe and classify mood swings.
**Session 5**	Explain, interpret, and describe the relationship between situation and emotion; awareness of situations and thoughts that relate to mood changes; describe thoughts, beliefs, concerns, perceptions, and concepts related to situations. **Assignment**: Note down support for the inaccuracy of “hot thoughts”. Gather evidence to confirm or reject a hot thought.
**Session 6**	Evaluating thoughts and challenging them; role playing in the group to reform and challenge automatic thoughts; analyzing behavioral changes in various situations; assessing personal responsibility; identifying main beliefs. **Assignment**: Test new experiences, experiments and practical plans.
**Session 7**	Identifying underlying principles and assumptions; therapeutic interventions; identifying worries and their benefits and harms; investigating ways to manage negative events of the past; identifying and classifying violent thoughts and emotions associated with them; mental imagery. **Assignment**: Fill out the Metacognitions Questionnaire
**Session 8**	Treatment of depression; cognitive restructuring; improving interpersonal relationships; timed schedule of activities; recording weekly activities; taking a lesson from these schedules; understanding anxiety ,its characteristics, and cognitive aspects; practicing relaxation, controlled breathing, visualization, and distraction; understanding anger, guilt and shame; anger restraint methods; assertiveness training; overcoming feelings of guilt.

**Table 2:** Relationship between psychological symptoms scores before and after of intervention in two groups (cognitive behavioral therapy and control)

**Group**	**Score on psychiatric symptoms**	**Mean and standard deviation**	**P value**
Cognitive-behavioral therapy	Before intervention	6/47±20/4	0/012*
	After intervention	5/68±16/1	
Control	Before intervention	4/42±21/5	0/552
	After intervention	8/03±19/6	

* Significant at 0.05 level

**Table 3:** Comparison of Mean and standard deviation of psychological symptoms of premenstrual syndrome in Cognitive-behavioral therapy group with control group

	**Cognitive-behavioral therapy** ** group**	**Control group**	**P value**
Mean and standard deviation before intervention	20/4±6/47	21/5±4/42	0/884
Mean and standard deviation after intervention	16/1±5/68	19/6±8/03	0/233

**Table 4:** Comparison of Cognitive-behavioral therapy and control group in terms Psychological symptoms of Premenstrual syndrome and social interferences scores

**Group **	**Social interferences score**	**Mean and standard deviation**	**P value**
Cognitive-behavioral therapy group	Before intervention	3/43±7/35	0/012
	After intervention	2/16±5/28	
Control	Before intervention	2/19±9/15	0/03
	After intervention	4/04±6/69	

*Significant at 0.05 level

**Table 5:** Relationship between age of participants and psychological symptoms of Premenstrual syndrome scores in before and after intervention

	**Age (correlation value)**	**P value**
Psychological symptoms score before intervention	0/070	0/771
Psychological symptoms score after intervention	-0/162	0/496

## Conclusion ‎

The results of the present study indicate that cognitive behavioral training can be a ‎useful skill for dealing with psychological symptoms caused by PMS. Other studies also ‎show that this kind of treatment is effective on the physical symptoms of PMS. CBT ‎gives female students new insight into themselves, their symptoms, and the individuals ‎around them, reinforcing their ability to identify solvable problems and helping them ‎become successful in better controlling their lives. This type of treatment is rarely ‎available, but it is the right choice for students suffering from PMS symptoms who ‎cannot tolerate drug therapy or for students who use non-prescribed medication.‎

## Limitation

Some limitations of the current study included the chance that some participants may ‎not express their problems and some samples might withdraw from active participation ‎in group therapy. The researchers attempted to minimize these limitations by using ‎counseling skills and providing their e-mail addresses and contact numbers to ‎participating students for special cases. A similar research with a long follow-up is ‎recommended to evaluate the medium-term and long-term effects of cognitive-‎behavioral group therapy.‎
